# Intra-arterial application of nimodipine in reversible cerebral vasoconstriction syndrome: a neuroradiological method to help differentiate from primary central nervous system vasculitis

**DOI:** 10.1186/s42466-022-00173-0

**Published:** 2022-02-28

**Authors:** Daniel Strunk, Roland Veltkamp, Sven G. Meuth, René Chapot, Markus Kraemer

**Affiliations:** 1 Department of Neurology, Alfried Krupp Hospital Essen, Alfried-Krupp-Straße 21, 45131 Essen, Germany; 2 Department of Brain Sciences, Imperial College London, London, UK; 3 Department of Neurology, University Hospital Düsseldorf, Heinrich-Heine-University Düsseldorf, Düsseldorf, Germany; 4 Department of Intracranial Endovascular Therapy, Alfried Krupp Hospital Essen, Essen, Germany

**Keywords:** Reversible cerebral vasoconstriction syndrome, Angiography, Nimodipine, Stroke

## Abstract

**Background:**

Reversible cerebral vasoconstriction syndrome (RCVS) is characterized by a prolonged, but self-limiting segmental cerebral vasoconstriction. Neurological outcomes vary, but can be severe. The clinical hallmark of RCVS is thunderclap headache, which might come along with further neurological symptoms. Distinguishing RCVS from other entities, such as primary angiitis of the central nervous system (PACNS), is of utmost importance for appropriate therapy. The angiographic response to intra-arterial nimodipine application has been suggested as an additional diagnostic criterion for RCVS but confirmatory studies are limited. We aimed to evaluate the angiographic nimodipine test.

**Methods:**

We reviewed retrospectively the clinical and imaging data of 13 RCVS patients, who were admitted to a single German neurological department between January 2013 and December 2020.

**Results:**

Out of 13 patients diagnosed with RCVS, 4 patients underwent an angiographic nimodipine test. In all 4 patients cerebral vasoconstriction completely resolved during nimodipine application. Among the four patients with a positive test, there was one individual, in whom a response was detected after a delay of 60 min. In all patients, we found a complete resolution of cerebral vasoconstriction within 12 weeks.

**Conclusion:**

Our findings support the usefulness of the application of nimodipine in diagnosing RCVS. Prolonged angiographic observation of the vascular response after nimodipine injection is important.

## Introduction

Reversible cerebral vasoconstriction syndrome (RCVS) is characterized by transient or long-lasting thunderclap headache as well as multi-focal segmental vasoconstriction of cerebral arteries. Due to recurrent watershed ischemic stroke, as well as cortical cerebral bleedings, misdiagnosis and false treatment bear the risk of persisting disability. The typical vascular abnormalities in RCVS usually resolve within twelve weeks. In the past, RCVS was erroneously assumed to be an inflammatory disorder, which is reflected by terms such as ‘Migraine angiitis’, ‘drug-induced Angiitis’, and ‘benign angiitis of the central nervous system’ [1]. The former term ‘Call Fleming Syndrome’ is of historical significance [2], whereas the current term ‘Reversible cerebral vasoconstriction syndrome’ summarizes the different historical entities, which vary only in different triggers (e.g. pregnancy or vasoactive drugs) of the prolonged, but reversible vasoconstriction.

**Fig. 1:**
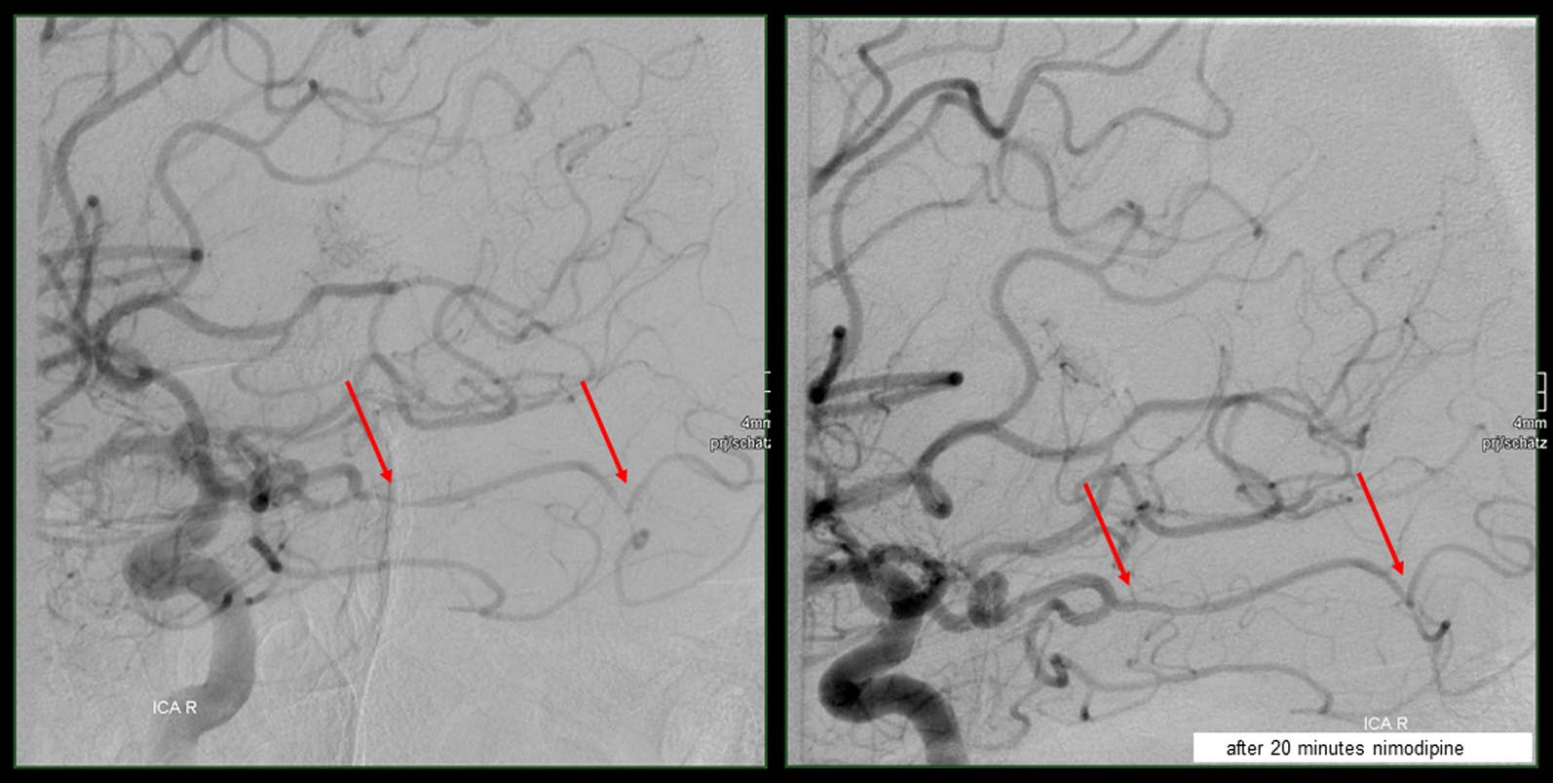
Angiogram demonstrating reversibility of intracranial stenoses after nimodipine

To some extent, diagnosis of this disorder has been facilitated by the current diagnostic criteria (see Table 1), published in 2007 [1]. Nonetheless, there are still remaining uncertainties and shortcomings. Given that the current diagnostic criteria have already been proven to be, at least partially, invalid in some cases of RCVS [3], making the correct diagnosis is challenging. This applies even more, as misinterpretation of (para-) clinical findings might lead to wrong or even adverse therapeutic measures, such as immunosuppressive treatment when RCVS is mistaken for  Primary angiitis of the central nervous system (PACNS). On the one hand, renouncing the treatment with oral nimodipine when PACNS is wrongly assumed, might result in (additional) cerebral infarctions, haemorrhages and a persisting increase in disability. On the other hand, a long-term therapy with immunosuppressants makes patients prone to infections and side effects, such as Cushing’s syndrome. In this context, the intra-arterial application of nimodipine in RCVS syndrome, first proposed by Linn et al. [4], turned out to be a helpful means to increase diagnostic accuracy by testing the reversibility of vasoconstriction during catheter angiography. Linn et al. examined a cohort of nine patients with suspected RCVS, in whom intra-arterial nimodipine was administered due to a deteriorating clinical status. Though not primarily striving for establishing a diagnostic tool for the differentiation between RCVS and other entities, the authors retrospectively concluded that their observations might serve as a basis for such a test. This is why, its parameters were not exclusively developed for diagnosing RCVS, but were derived from the local standards for the treatment of vasospasms related to subarachnoid haemorrhage. This applies, among others, to the duration of the procedures. Despite the retrospective character of the findings of Linn et al., they could demonstrate a reversibility of vasoconstriction in a cohort of nine patients with suspected RCVS within an observation period of up to one hour. Due to the rarity of RCVS being suspected by clinicians, as well as the rare implementation of this diagnostic test, it has not been verified in its reliability (Fig. 1). Moreover, the experience of the previous years shows deviations from this scheme, i.e. stopping the test after less than 60 min, potentially resulting in misdiagnosis.

**Table 1:** Critical elements for the diagnosis of RCVS. Adapted from Calabrese et al. [1]

*Clinical features*
Severe, acute ‘thunderclap’ headaches, with or without additional neurological signs due to (watershed) strokes, subarachnoidal haemorrhages
Associated conditions (e.g. medication, drugs, and blood products)
*Radiological features*
Conventional angiography or indirect CTA or MRA with ‘vasculitis-like’ multi-focal segmental cerebral artery vasoconstriction
Watershed (strokes)
Subarachnoidal haemorrhages (cortical)
No evidence for aneurysmal subarachnoid haemorrhage
Reversibility of angiographic abnormalities within 12 weeks after onset
*Others*
Normal or near-normal cerebrospinal fluid analysis (protein level < 80 mg%, leucocytes < 10 mm^3^, normal glucose level)

CTA, computed tomographic angiography; MRA, magnetic resonance angiography

Our study aimed to re-evaluate current diagnostic criteria and common diagnostic tools taking particular account of the intra-arterial application of nimodipine.

## Methods

We retrospectively reviewed the clinical and paraclinical data of 13 RCVS patients who were admitted to the Department of Neurology of the Alfried Krupp-Hospital in Essen (Germany) from January 2013 to December 2020. Given that there is no specific code for RCVS, we reviewed the case records of 135 patients who were encoded as ‘Other cerebrovascular diseases’ (I67.0), according to the International Classification of Diseases (ICD-10). Fourteen patients fulfilled the criteria of typical clinical signs of RCVS, i.e. headache of high or even maximum intensity plus further neurological symptoms, and cerebral vasoconstriction demonstrated by computed tomography (CT), magnetic resonance (MR), digital subtraction angiography (DSA), and transcranial ultrasound. One patient was excluded due to persistent cerebral arterial stenosis, finally resulting in the diagnosis of PACNS. In the remaining 13 patients, the diagnosis ‘RCVS’ was made with reference to the aforementioned diagnostic criteria [1]. For every patient, a standardized set of clinical, imaging and laboratory data was collected by retrospective chart review. In particular, information on demographics, medical history, initial clinical presentation, laboratory analysis, and neuroimaging were gathered. All included patients underwent neurological examinations at least twice, at symptom onset and approximately three months later. The angiographic procedure with nimodipine test was described by Linn et al. [4] and is summarized in Table 2. As nimodipine (Nimotop®S, 10 mg/50 ml, BayerVital GmbH, Leverkusen, Germany) is only medically approved intravenously in vasospasms after subarachnoidal cerebral bleedings, all intra-arterial angiographic procedures represent an off-label use. In all patients, normalisation of caliber irregularities was judged just by eye. Vessel diameter was not measured exactly before and after nimodipine due to practical reasons.

**Table 2:** Description of technique for angiographic nimodipine test

Insertion of a 4F sheath in Seldinger technique establishing a right femoral artery access under local anaesthesia
Diagnostic cerebral angiography from internal carotid arteries bilaterally, as well as from vertebral artery
If suspicious caliber irregularities are found in standard projections, acquisition of additional projections (e.g. 45° oblique)
Leave 4 F catheter (Tempo 4 F, VER 135°, Cordis Corporation, Miami Lakes, USA) in internal carotid or vertebral artery on the side of most prominent caliber irregularities
Dissolve 3 mg (15 ml) of Nimodipine (Nimotop®S, 10 mg/50 ml, BayerVital GmbH, Leverkusen, Germany) in 1000 ml isotonic NaCl
Infuse nimodipine at a rate of 3 mg per hour (via 3-way stopcock)
In case of known low blood pressure continous blood pressure monitoring is advisable
Take pictures in the same projection after 15, 30, 45 and 60 min. Patient's head should be fixed to ensure constant projection
Withdrawal of catheter and compression of the groin

## Results

### Demographic and clinical data

Thirteen patients were identified to suffer from RCVS (ten females, three males, mean age = 47 years, range 18–74 years). In three patients, medical history pointed to a link between the use of antidepressants or drug consumption. In one patient with relapsing remitting multiple sclerosis, a causal association with the disease modifying therapy with fingolimod was assumed [5]. Focussing on initial clinical presentation of RCVS, all patients suffered from headache, which did not necessarily reach the intensity of severe, acute ‘thunderclap’ headache in all cases. Other demographic and clinical data are shown in Table 3.

**Table 3:** Clinical characteristics

*Demographics*	
Age (mean ± standard deviation) (year)	47.1 ± 15.8
Female/male	10/3
*Medical history, n (%)*	
Hypertension	5 (38.5)
Tobacco use	4 (30.8)
Migraine	1 (7.7)
Depression/anxiety	2 (15.4)
Vasoactive drug or medication	3 (23.1)
*Initial clinical presentation, n (%)*	
Thunderclap headache	8 (61.5)
Other headache	5 (38.5)
Motor deficit	8 (61.5)
Sensory disorder	4 (30.8)
Speech disorder	2 (15.4)
Cognitive disorder	1 (7.7)
Visual/acoustic symptoms	4 (30.8)
Vegetative symptoms	5 (38.5)
Dizziness/vertigo	1 (7.7)
Impaired consciousness	2 (15.4)

### Laboratory data

Eleven of thirteen patients underwent lumbar puncture close to the time of symptom onset. In five of these patients (45.5%), CSF leukocyte count exceeded the value of 10/mm^3^ (11–29/mm^3^), included in the current diagnostic criteria. Irrespective of this threshold value, which was established to facilitate the differentiation between RCVS and inflammatory vasculopathies, abnormal CSF results were found in nine individuals (81.8%). Conversely, CSF analysis revealed the presence of oligoclonal bands in a minority of patients, two of whom were already diagnosed with multiple sclerosis [5]. Other paraclinical data are summarized in Table 4.

**Table 4:** Laboratory and neuroimaging

*Laboratory analysis*	
ESR (mean ± standard deviation) (mm/h)	15/28 ± 8/14 (n = 4)
CRP abnormal (≥ 0.5 mg/dl), n (%)	2 (15.4)
Vasculitis parameters positive, n (%)	2 (25)
Abnormal CSF, n (%) 9 (81.8)	
Cell count (mean; median; range) (cells/mm^3^)	10.3; 8.5; 1–29 (n = 10)
Protein concentration (mean; median, range) (mg/l)	456.4; 429.5; 363–604 (n = 8)
Oligoclonal bands positive, n (%)	3 (27.3)
*Neuroimaging, n (%)*	
Infarct	6 (46.2)
Border zone infarct	2 (28.6)
Territorial infarct	4 (57.1)
Cerebellar infarct	1 (14.2)
Multiple infarcts	4 (30.8)
Microbleeds	1 (7.7)
Parenchymal hemorrhage	1 (7.7)
Subarachnoid hemorrhage	4 (30.8)
Cortical localisation	3 (75)
Posterior reversible encephalopathy syndrome (PRES)	2 (15.4)
Abnormal CTA/MRA	6 (54.5)

### Neuroimaging

The most frequent findings in computed tomography and magnetic resonance imaging were cerebral infarction with border zone infarcts being outnumbered by territorial infarcts and intracranial haemorrhage, most frequently cortical subarachnoid haemorrhage. A majority of patients with ischaemic stroke suffered multiple infarctions. Posterior reversible encephalopathy syndrome (PRES) was documented in two individuals.

Segmental multifocal cerebral vasoconstriction was found in all subjects by means of neurovascular ultrasound, magnetic resonance angiography or conventional angiography. Vascular abnormalities were shown to be completely regressive in follow-up imaging after three months or following intraarterial nimodipine application. DSA was performed for diagnostic reasons in all patients. In those patients, who were primarily admitted to our institution, and who fulfilled the diagnostic criteria of RCVS, as defined by Calabrese et al. [1], we additionally administered intra-arterial nimodipine. Further patients were examined because external hospitals asked for a second opinion (n = 5). In these cases, PACNS was suspected by our colleagues. In contrast to previous pathologic results, angiographic findings had already normalized, when the respective patients were examined in our hospital. Consequently, we renounced nimodipine tests. However, diagnostic criteria of RCVS were fulfilled, which allowed us to rule out PACNS and diagnose RCVS. In two cases DSA was performed when oral nimodipine intake had already been initiated in advance due to the detection of subarachnoid haemorrhage or a severe disease onset with coma. In another patient, clinical signs, including thunderclap headache, and MRI findings were typical of RCVS, while subsequent DSA was normal. In this case, we hypothesized that the pathological conditions might have been the result of a went-through RCVS. A possible differential diagnosis would have been a migrainous infarction, representing a rare condition, too. Finally, the last patient of our cohort was initially diagnosed with PACNS, which was revised when multifocal stenoses declined completely within three months. This seems to be conclusive all the more, given that CSF parameters and brain biopsy were unremarkable.

**Fig. 2:**
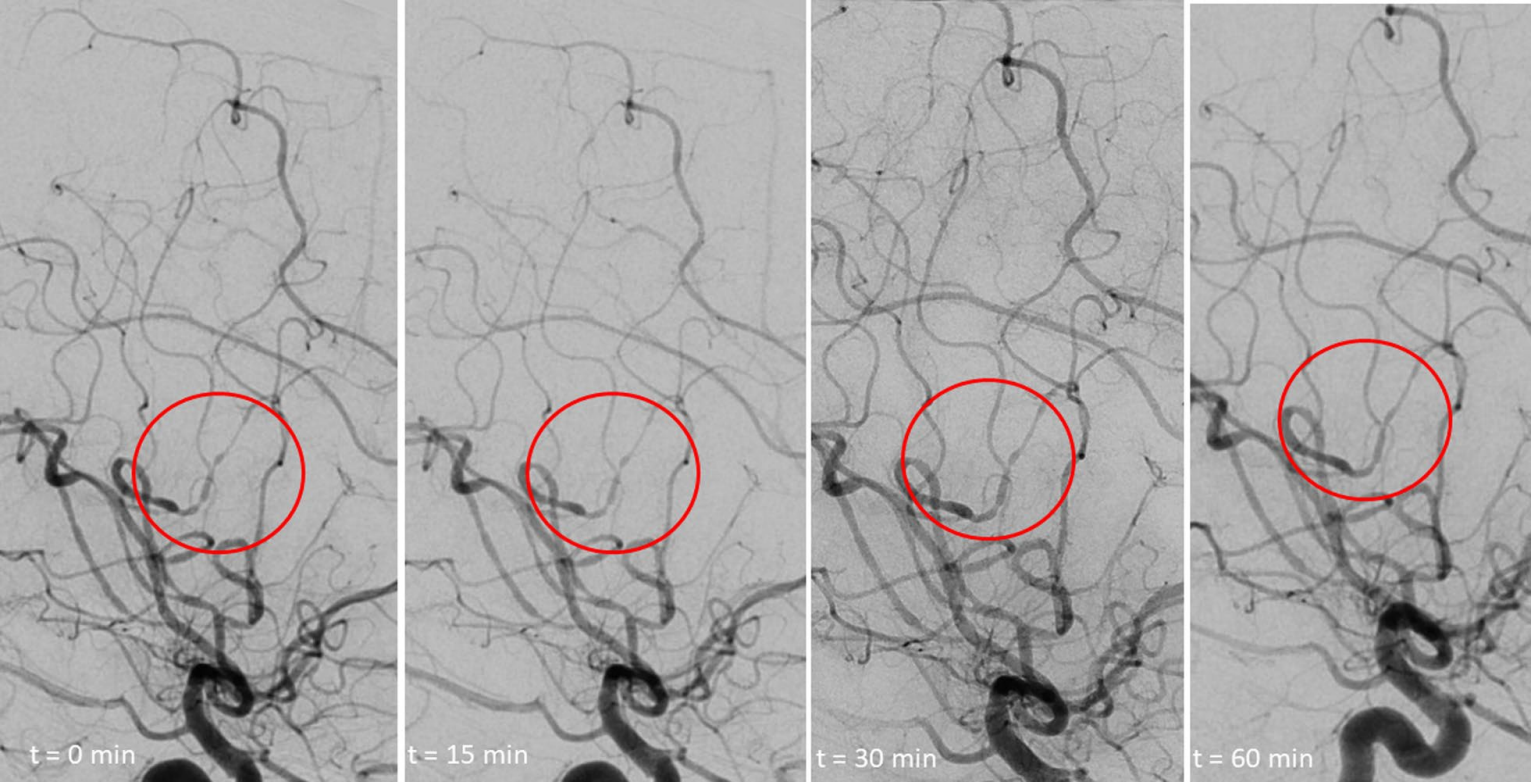
Angiogram showing delayed reversibility of intracranial stenoses after nimodipine

Out of thirteen patients who underwent conventional angiography, four were examined by means of intraarterial application of nimodipine. Time to reversal of multifocal cerebral vasoconstriction was 15, 20, 25 and 60 min (mean duration 30 min) (see Fig. 2). In one additional patient, a negative test had to be stopped due to an allergic reaction. Finally, as outlined in the Discussion section, the diagnosis ‘PACNS’ was made. Further cases of a final diagnosis of PACNS are not included in our analysis, because they were excluded from further work-up after searching our database for records, which were encoded with the ICD-10 code I67 (other cerebrovascular diseases).

## Discussion

Despite scientific and technological progress since the description of RCVS by Call et al.[2] with its characteristic symptoms and abnormal angiogram findings in 1988, diagnosing RCVS remains challenging. This is because its differential diagnoses are not simple to rule out, even when a typical “string and beads” appearance in cerebral angiography is present. At an early stage, other entities characterized by similar imaging and clinical features, especially primary and secondary vasculitis of the central nervous system, are the most relevant differential diagnoses [6, 7]. Unfortunately, though the sensitivity of diagnostic angiography is high, specificity is insufficient. Thus, further tools of differentiation are required.

Against this background, our analysis of (para-) clinical attributes of 13 patients yields the following main findings:

(1) Intraarterial application of nimodipine in the context of RCVS facilitates diagnosing or ruling out the disease; (2) Being patient and continuing the procedure for up to 60 min is worth the effort, given that, in some cases, reversibility of vasoconstriction is detected at a late stage; (3) Therefore, stopping the test earlier, bears the risk of missing the correct diagnosis and taking adverse therapeutic measures; (4) Using the test as initially proposed, i.e. for a period of time of 60 min, in everyday medical care is recommendable (see Fig. 1).

According to our experiences, in contrast to RCVS, which is characterized by vasospastic changes in vessel diameter, the inflammatory pathophysiology of intracranial stenoses in PACNS leads to a missing effect during intra-arterial nimodipine application. Despite general dilatation of all vessels induced by nimodipine, the proportion of stenoses is unchanged in PACNS. A good example of this observation is the case of a fifty-year-old male patient, who presented with multiple cerebral infarctions, severe headache, and an inconclusive brain biopsy. Intra-arterial nimodipine application failed to normalize the vessel status, characterized by multifocal beaded stenoses. Finally, PACNS was diagnosed, which was also supported by the finding of a CSF pleocytosis. Angiographic findings were improved six months after immunosuppressive treatment, but marked stenoses were still evident, which contradicts the definition of RCVS.

Valuable additions to Linn’s retrospective observations have been made by Sequeiros et al. [8], who showed that measurement of the change in the arterial calibers after intra-arterial verapamil can even enhance diagnostic accuracy.

Apart from that, demographic data, initial symptoms and angiographic signs basically corresponded to the already existing literature, whereas migraine was a rather rare comorbidity in our cohort [6]. As already reported by our group in 2018, the majority of patients had abnormal results in CSF analysis [3]. This is why we question the threshold of < 10/mm^3^ CSF leukocytes to differentiate from cerebral vasculitis [1]. This notion is supported by Ducros et al. [6], who reported leukocyte levels up to 35/mm^3^ in individuals with RCVS. Consequently, we suggest not to rely blindly on the aforementioned threshold, but to consider the complete context.

The significance of different (para-) clinical features for an accurate differentiation between RCVS and PACNS has already been described by de Boysson et al. [7] in a large national cohort. Furthermore, it has already been demonstrated that vessel wall imaging does not only show abnormalities in patients with PACNS, but might also be found in RCVS, complicating differential diagnostics even more [9].

In contrast to CSF analysis, we found inflammation markers in the blood, i.e. elevated ESR and CRP, vasculitis parameters, and oligoclonal bands to be negative in most cases of RCVS. This might be helpful in differentiating RCVS from secondary vasculitis of the central nervous system, but does not allow a clear distinction from PACNS. Concerning neuroimaging, the diagnostic criteria include the terms ‘(watershed) strokes’ and ‘(cortical) subarachnoid haemorrhages’. Our data shows that other types of infarction or haemorrhage might occur as well, which requires physicians to have an open mind and to be familiar with the different phenotypes of RCVS.

Altogether, a clear and early distinction of RCVS from its differential diagnoses, especially PACNS, is crucial. This analysis confirmed the reliability of a 60-min-angiographic nimodipine test, which is in line with the retrospective findings reported by Linn et al. and argues for a wider application in daily practice to help differentiate RCVS and other vasopathies, for example PACNS.

Our study has strengths and limitations. Striving for a balanced analysis, we point out the usefulness and pitfalls of intraarterial application of nimodipine at the same time. The composition of our cohort is comparable to the initial study by Linn et al. Furthermore, we took a variety of (para-) clinical parameters into account in order to give a comprehensive characterisation of the patients under investigation. Nonetheless, the number of included patients suffering from the rare disease ‘RCVS’ and having undergone intraarterial nimodipine application is still low. Moreover, the selectivity of this neuroradiological method is not proven, although it is plausible regarding the pathophysiology of the disease. Another limitation is that the changes in diameter before and after nimodipine were judged just by eye due to practical reasons. A prospective study to validate these findings is not feasible due to the difficulties in differentiating these cohorts and the rarity of the disease. Taking this into account, this neuroradiological method can only be interpreted in the context of all other clinical and anamnestic findings. RCVS can only be proven if the angiographic findings are fully remitted after 12 weeks.

Nevertheless, our study provides some clues how RCVS can and should be diagnosed, enabling physicians to make an early diagnosis and start an appropriate treatment at an early stage.

## Conclusion

Our findings support the usefulness of intra-arterial nimodipine as a potential diagnostic tool in patients with headache and angiography suspected to be “typical for vasculitis”. Far-reaching therapeutic decisions, which require the greatest possible clarification in differentiating RCVS from other neurological disorders with comparable clinical and angiographic characteristics, can be based on this test. In this context, it is paramount to carry out the test for 60 min as not to miss late changes in vessel diameter.

Our data support and complement the preliminary findings of Linn et al. We recommend the intra-arterial nimodipine test or oral treatment with nimodipine with consequent follow-up for twelve weeks as a safe strategy in patients with acute onset of severe headache, and angiographic signs of beaded stenosis.

## Data Availability

Data transparency.

## References

[1] Calabrese LH, Dodick DW, Schwedt TJ, Singhal AB (2007). Narrative review: Reversible cerebral vasoconstriction syndromes. Annals of Internal Medicine.

[2] Call GK, Fleming MC, Sealfon S (1988). Reversible cerebral segmental vasoconstriction. Stroke.

[3] Kraayvanger L, Berlit P, Albrecht P (2018). Cerebrospinal fluid findings in reversible cerebral vasoconstriction syndrome: A way to differentiate from cerebral vasculitis?. Clinical and Experimental Immunology.

[4] Linn J, Fesl G, Ottomeyer C (2011). Intra-arterial application of nimodipine in reversible cerebral vasoconstriction syndrome: A diagnostic tool in select cases?. Cephalalgia.

[5] Kraemer M, Weber R, Herold M, Berlit P (2015). Reversible cerebral vasoconstriction syndrome associated with fingolimod treatment in relapsing-remitting multiple sclerosis three months after childbirth. Multiple Sclerosis.

[6] Ducros A, Boukobza M, Porcher R (2007). The clinical and radiological spectrum of reversible cerebral vasoconstriction syndrome. A prospective series of 67 patients. Brain.

[7] de Boysson H, Parienti J-J, Mawet J (2018). Primary angiitis of the CNS and reversible cerebral vasoconstriction syndrome: A comparative study. Neurology.

[8] Sequeiros JM, Roa JA, Sabotin RP (2020). Quantifying intra-arterial verapamil response as a diagnostic tool for reversible cerebral vasoconstriction syndrome. AJNR: American Journal of Neuroradiology.

[9] Mossa-Basha M, Hwang WD, De Havenon A (2015). Multicontrast high-resolution vessel wall magnetic resonance imaging and its value in differentiating intracranial vasculopathic processes. Stroke.

